# Radiogenomics of breast cancer using dynamic contrast enhanced MRI and gene expression profiling

**DOI:** 10.1186/s40644-019-0233-5

**Published:** 2019-07-15

**Authors:** Albert C. Yeh, Hui Li, Yitan Zhu, Jing Zhang, Galina Khramtsova, Karen Drukker, Alexandra Edwards, Stephanie McGregor, Toshio Yoshimatsu, Yonglan Zheng, Qun Niu, Hiroyuki Abe, Jeffrey Mueller, Suzanne Conzen, Yuan Ji, Maryellen L. Giger, Olufunmilayo I. Olopade

**Affiliations:** 1 Department of Hematology/Oncology, University of Chicago, 900 East 57th Street, KCBD 8100, Chicago, IL 60637 USA; 2 Department of Radiology, University of Chicago, 5841 South Maryland Avenue, MC2026, Chicago, IL 60637 USA; 3 Program for Computational Genomics and Medicine, NorthShore University Health System, 1001 University Pl, Evanston, IL 60201 USA; 4 Department of Pathology, University of Chicago, Chicago, IL USA

**Keywords:** Radiogenomics, Imaging genomics, Breast cancer

## Abstract

**Background:**

Imaging techniques can provide information about the tumor non-invasively and have been shown to provide information about the underlying genetic makeup. Correlating image-based phenotypes (radiomics) with genomic analyses is an emerging area of research commonly referred to as “radiogenomics” or “imaging-genomics”. The purpose of this study was to assess the potential for using an automated, quantitative radiomics platform on magnetic resonance (MR) breast imaging for inferring underlying activity of clinically relevant gene pathways derived from RNA sequencing of invasive breast cancers prior to therapy.

**Methods:**

We performed quantitative radiomic analysis on 47 invasive breast cancers based on dynamic contrast enhanced 3 Tesla MR images acquired before surgery and obtained gene expression data by performing total RNA sequencing on corresponding fresh frozen tissue samples. We used gene set enrichment analysis to identify significant associations between the 186 gene pathways and the 38 image-based features that have previously been validated.

**Results:**

All radiomic size features were positively associated with multiple replication and proliferation pathways and were negatively associated with the apoptosis pathway. Gene pathways related to immune system regulation and extracellular signaling had the highest number of significant radiomic feature associations, with an average of 18.9 and 16 features per pathway, respectively. Tumors with upregulation of immune signaling pathways such as T-cell receptor signaling and chemokine signaling as well as extracellular signaling pathways such as cell adhesion molecule and cytokine-cytokine interactions were smaller, more spherical, and had a more heterogeneous texture upon contrast enhancement. Tumors with higher expression levels of JAK/STAT and VEGF pathways had more intratumor heterogeneity in image enhancement texture. Other pathways with robust associations to image-based features include metabolic and catabolic pathways.

**Conclusions:**

We provide further evidence that MR imaging of breast tumors can infer underlying gene expression by using RNA sequencing. Size and shape features were appropriately correlated with proliferative and apoptotic pathways. Given the high number of radiomic feature associations with immune pathways, our results raise the possibility of using MR imaging to distinguish tumors that are more immunologically active, although further studies are necessary to confirm this observation.

**Electronic supplementary material:**

The online version of this article (10.1186/s40644-019-0233-5) contains supplementary material, which is available to authorized users.

## Background

Recent advances in image acquisition, computational power, and algorithmic development have increased the awareness of computational image analysis methods, allowing for computer-extracted features, i.e. phenotypes, from computer-aided diagnosis and quantitative imaging algorithms to yield “radiomics”, i.e., the high throughput conversion of complex imaging data sets into a multi-dimensional feature space with extractable characteristics [1–3]. Imaging techniques can provide extractable information about the tumor non-invasively and have been shown to provide information about the underlying histopathology [4–9] as well as genetic makeup of the tumor and tumor microenvironment [10–13]. Adoption of this technology in an effort to phenotypically characterize solid tumors have been performed primarily in patients with glioblastomas, head and neck, lung, and breast cancers [10, 12, 14–26].

Correlating specific image-based phenotypes (radiomics) with large-scale genomic analyses (genomics) is an emerging area of research commonly referred to as “radiogenomics” or more specifically “imaging-genomics”. This emerging field addresses novel high-throughput methods of associating information-rich radiographic images with genomic data as well as other clinically relevant information [2, 12, 13, 21–25, 27]. Radiogenomics has the potential to impact diagnostic and therapeutic strategies by creating more individualized prognostic signatures and real time measurements in response to therapy.

The application of radiogenomics to breast cancer has recently been gaining more attention. For example, studies using mammographic images showed that radiographic texture analysis could identify patients who are more likely to carry *BRCA1/2* mutation [28], and parenchymal pattern and breast density were associated with *UGT2B* gene variation [29]. Higher-dimensional imaging data such as magnetic resonance imaging (MRI) have also been used to associate radiologic features with underlying clinical or histological characteristics [12, 13, 16, 17, 30–34]. However, most of these studies have focused on a few clinical, histopathologic, or genetic features. For example, Bhooshan et al. classified features that were correlated with the ability to distinguish invasive vs. non-invasive lesions as well as with tumor grade [12, 13]. Mazurowski et al. demonstrated a relationship between MRI enhancement dynamics with the luminal subtypes of breast cancer [33]. Agrawal et al. was able to show a correlation between certain MRI phenotypes and HER-2 receptor subtype [16]. Gene expression profiling was examined to a limited degree in another study that correlated MRI phenotypes with Oncotype DX output that predicted for survival [31].

Recent studies conducted by the TCGA Breast Phenotype Group examined breast MRI radiomics with clinical stage, molecular classification, risk of recurrence, gene expression profiling, and other genomic analyses [21–25]. In the most comprehensive breast radiogenomics study to date, they analyzed 91 breast cancer cases that were extracted from The Cancer Genome Atlas (TCGA) and The Cancer Imaging Archive (TCIA) datasets. In that study, MRI tumor phenotypes derived from 1.5 Tesla scanners were examined in relation to genomic features including DNA mutation, copy number variation, miRNA expression, protein expression, and gene expression profiles. Their data showed that gene expression profiling provided the most robust information in terms of correlation with radiomic phenotypes [25].

Here, we sought to further elucidate and validate relationships between imaging phenotypes and gene expression pathways by analyzing clinical MRI data of breast cancers taken from higher resolution 3 T scanners at the University of Chicago Medical Center using RNA sequencing transcriptomic data extracted from corresponding frozen tissue samples.

## Materials and methods

### Sample size and selection

Case selection was initially derived from a pool of 1236 patients with breast cancer who had undergone surgery between 2007 and 2012. All patients had IRB consent to allow for use of their tissue samples for further research. We limited our search to 226 potential cases after including only patient cases with frozen tissue available and those that had breast MRI performed prior to surgery for their breast cancer. We further excluded patients with only DCIS, those with documented *BRCA1/2* mutations, and those who had received neoadjuvant chemotherapy, as we were interested in pre-treatment gene expression and imaging profiles. Out of the remaining 139 cases, we selected 50 for this study, which included only images taken from our institution’s Phillips 3 T MRI scanner in order to reduce image acquisition variability. Forty-seven of these cases had complete dynamic contrast enhanced time-lapsed images, while three cases only had a single pre and post-contrast image. These 47 cases used for further analyses included 12 triple negative breast cancer cases as well as 26 randomly selected ER positive cases, 4 ER/HER2 positive cases, and 5 ER negative, HER2 positive cases. Three pairs of cases were derived from bilateral breast cancers, and two cases represented separate biopsy areas from the same tumor. (Full case characteristics included in Additional file 2: Figure S1).

### RNA extraction, sequencing, and expression quantification

Areas of malignant tissue were identified through light microscopy using representative top slides derived from 5 μm sections of frozen tumor samples. These areas were marked and scraped off for RNA extraction and purification, which was performed using the Qiagen AllPrep DNA/RNA/Protein mini kit protocol. All samples were treated with the optional DNase step as described in the protocol, and quality control was performed with the Agilent 4200 Tapestation system. cDNA libraries were constructed using the Illumina Truseq Stranded Total RNA with Ribo-Zero Human kit. RNA sequencing using 100 bp paired-end reads was performed on the Illumina HisSeq 4000 at a depth of 80 million reads per sample. Adapter sequences were removed from raw sequencing reads using Trimmomatic [35], a flexible trimmer designed for Illumina sequence data. Alignment was performed using the Spliced Transcripts Alignment to a Reference (STAR) software [36], and expression quantification was achieved using the python library HTSeq [37].

### DCE-MRI data acquisition

Dynamic contrast–enhanced MR images (DCE-MRI) were acquired using a dedicated 16-channel Philips SENSE-16 M breast coil on a 3 T Philips Healthcare Achieva system (Best, Netherlands). The DCE-MRI protocol acquires one pre- and 5 post-contrast-enhanced axial T1-weighted images with fat suppression using 3D gradient-echo sequences (TR/TE 5.0/2.5, flip angle 10°, acquisition matrix 448 X 448, slice thickness 1.6 mm, 250 slices, FOV 340 mm, acquisition time 75 s). Gadodiamide contrast material of 0.1 mmol/kg (Omniscan, GE Healthcare, Milwaukee, WI) was injected intravenously followed by a 20-mL saline flush at 2 mL/s. (Sample MRI images included in Additional file 3: Figure S2).

### Quantitative radiomics

The radiomics dataset had been calculated from MRIs in a de-identified fashion [12, 18–20, 24, 32]. Out of the 50 cases, there were 47 MRI cases available with full dynamic contrast enhanced images and 3 cases with only a single pre- and post-image available. Each MRI case had been reviewed by experienced academic radiologists at the University of Chicago. The primary tumor location was indicated by one of the radiologists (HA) and served as the only input to our quantitative image analysis MRI workstation for the subsequent quantitative radiomics calculations (Additional file 4: Figure S3). Each primary breast tumor was automatically segmented from background parenchyma using a fuzzy c-means clustering method [38]. Next, a total of 38 mathematical features of the breast tumors were automatically extracted. These features can be divided into six MRI phenotypic categories describing the tumor [1] size, [2] shape, [3] morphology, [4] enhancement texture, [5] kinetic curve assessment, and [6] enhancement-variance kinetics [12, 18–20, 24, 32, 39] (Additional file 5: Figure S4). The radiomic features were normalized to zero mean unit variance prior to downstream computerized analysis.

### Gene set enrichment analysis

We studied the associations between the transcriptional activities of genetic pathways included in the Kyoto Encyclopedia of Genes and Genomes (KEGG) database [40] and the tumor radiomic phenotypes using Gene Set Enrichment Analysis (GSEA) [41]. We used the GSEA tool implemented in Bioconductor R package PIANO [42] and the gene sets were KEGG pathways collected in the Molecular Signature Database [43] that includes 186 genetic pathways or modules covering a wide range of genetic and molecular functionalities. The RNA-seq data were filtered to remove the genes with unreliable expressions that could introduce significant noise or bias to the analysis results. A gene was excluded from the analysis, if its normalized read count was zero in 10 or more samples or its average read count over all samples was smaller than eight. After filtering, 15,544 genes were kept.

For each of the 38 radiomic phenotypes, we performed the GSEA for identifying the KEGG pathways whose transcriptional changes associated with the change of radiomic phenotype. The gene-level statistic used to characterize the relationship between a gene’s expression and a radiomic phenotype was the correlation coefficient resulted from the Spearman rank correlation test. Nominal *p*-value for evaluating the statistical significance of an association was calculated based on 10,000 random gene sets. The False Discovery Rate (FDR) was controlled for each radiomic phenotype over its association tests with all KEGG pathways using the Benjamini-Hochberg procedure [44]. An association was deemed as statistically significant if the adjusted *p*-value was ≤0.05. The associations between gene sets and radiomic phenotypes were tested separately for two different directions, i.e. positive association and negative association.

## Results

**Fig. 1:**
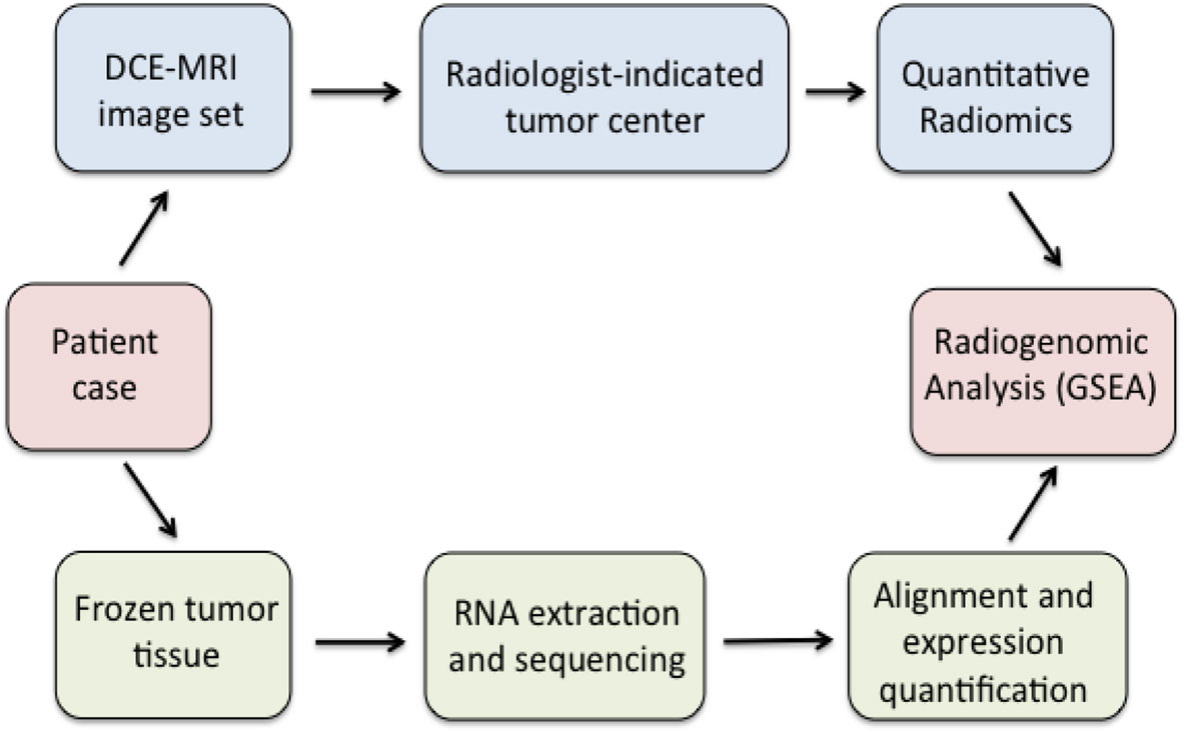
Schematic of radiogenomic pipeline and analysis. From each case we obtained fresh frozen tumor tissue as well as the corresponding set of dynamic contrast-enhanced MRI images of that tumor. Frozen tissue was then processed for total RNA extraction, sequencing, and expression quantification while the images were subjected to quantitative radiomic analysis after a staff radiologist indicated the tumor center for input. Final radiogenomic analysis combined these two datasets for gene set enrichment analysis

**Fig. 2:**
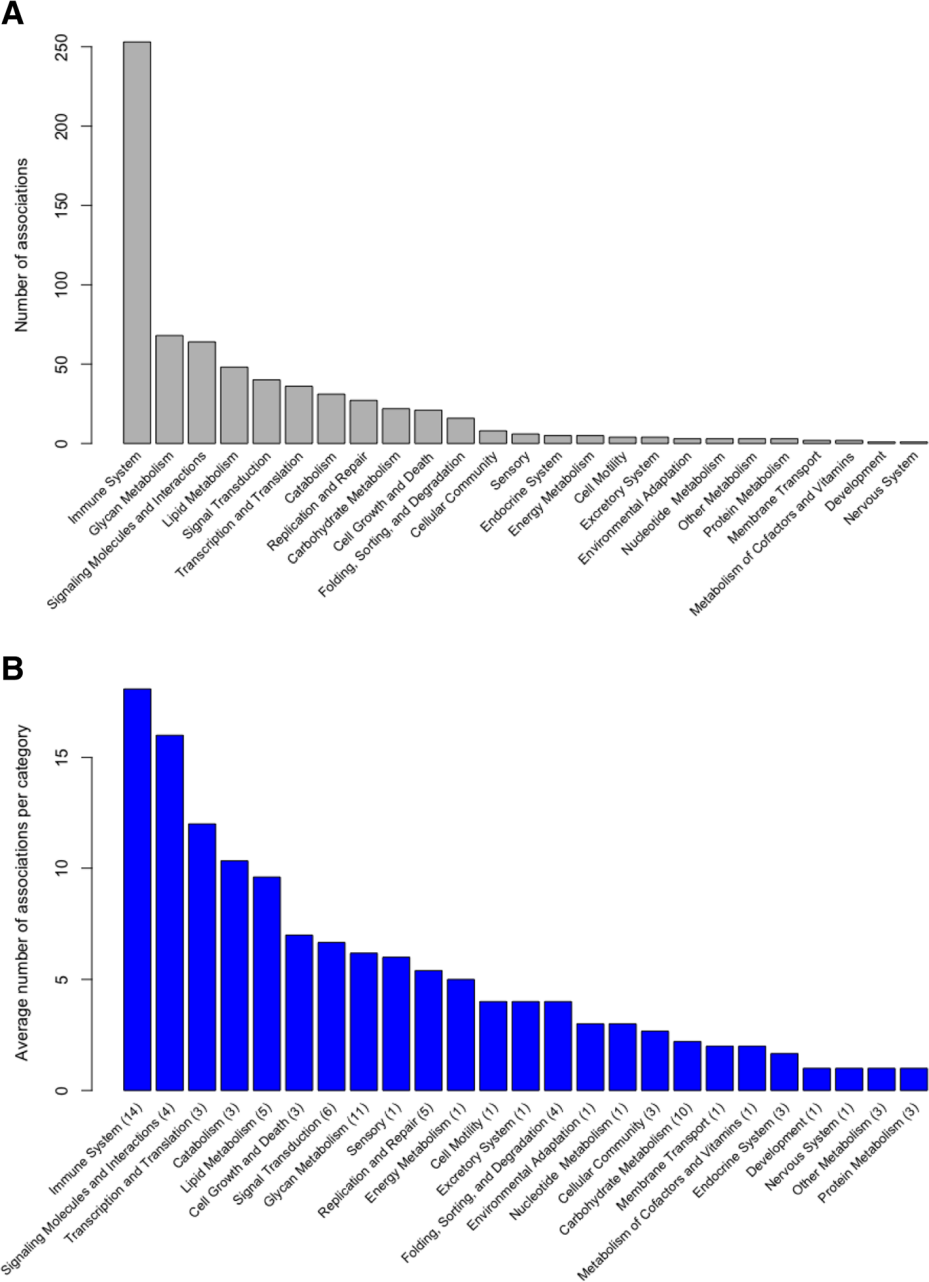
Radiomic feature associations by major pathway categories. Twenty-six major pathway categories pertaining to basic cellular and physiologic functions are denoted on the x-axis. **a** The total number of radiomic to pathway feature associations for each category is shown on the y-axis and graphed in descending order of frequency. **b** The average number of radiomic feature associations per pathway for each category is shown on the y-axis, with the number of pathways in each categories labeled in parenthesis on the x-axis

We successfully obtained radiomic and gene expression data from 47 cases (Fig. 1). Analysis of clinical DCE-MRI images of these cases along with corresponding gene expression data derived from RNA sequencing revealed 119 gene expression pathways that had at least one significant association with a radiomic phenotype (Additional file 1: Table S1). Several of these pathways were disease-specific pathways for particular organ systems, such as cardiovascular or neurological disease associations. As we were interested in basic cellular and physiologic pathways, we proceeded to analyze only non disease-specific pathways, which resulted in a total of 91 expression pathways representing 26 major categories, as denoted in the KEGG database (Additional file 1: Table S1). To analyze which pathway categories had the largest number of associations with radiomic phenotypes, we examined the average number of significant associations per pathway according to each of the 26 major categories (Fig. 2). The most heavily represented category involved immune regulation pathways. Other pathways that were also highly represented included those involved in cytokine-cytokine and cell adhesion receptor signaling, cellular proliferation and growth, lipid and glycan metabolism, and canonical signaling cascades including the JAK/STAT and VEGF pathways. We examine several of these major categories in further detail.

### Cell growth and death

**Fig. 3:**
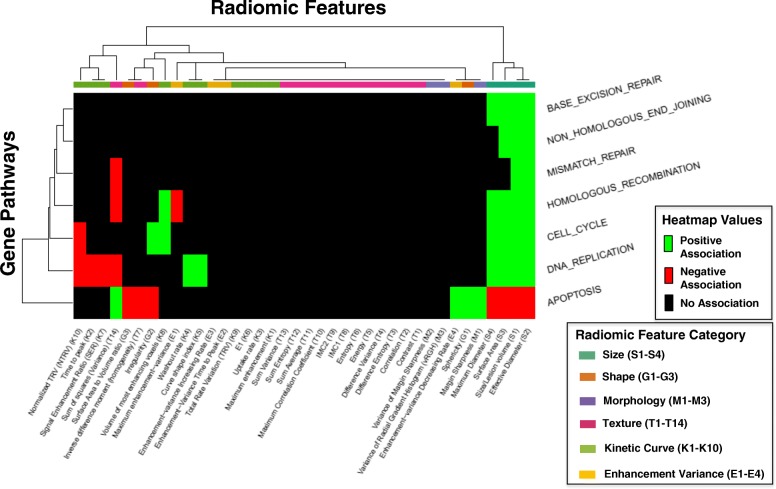
Cell Growth and Death**.** Associations between gene pathways and radiomic features are depicted via hierarchical clustering. Pathways involved in replication and proliferation that have at least one significant positive (green) or negative (red) association with a radiomic features are included in this diagram. These pathways include the canonical cell cycle, DNA replication, base excision repair, non-homologous end joining, mismatch repair, and homologous recombination. Radiomic features are classified by the six characteristic categories, including size, shape, morphology, enhancement texture, kinetic curve assessment, and enhancement-variance kinetics. The apoptosis pathway was also included for contrast

We first examined the relationship of radiomic features to gene expression pathways involved in replication, proliferation, and apoptosis (Fig. 3). Based on prior studies, we hypothesized that replication and proliferation pathways would be positively correlated with size features [25]. We included the apoptosis pathway as we expected it to offer a negative contrast. In support of this hypothesis, our results show that all size features (S1-S4) were positively associated with multiple replication and proliferation pathways and were negatively associated with the apoptosis pathway. Of note, all shape features (G1-G3) were also significantly associated with the apoptosis pathway. Sphericity was positively correlated to apoptosis while surface area to volume ratio and irregularity were negatively correlated to apoptosis.

### Immune pathways

**Fig. 4:**
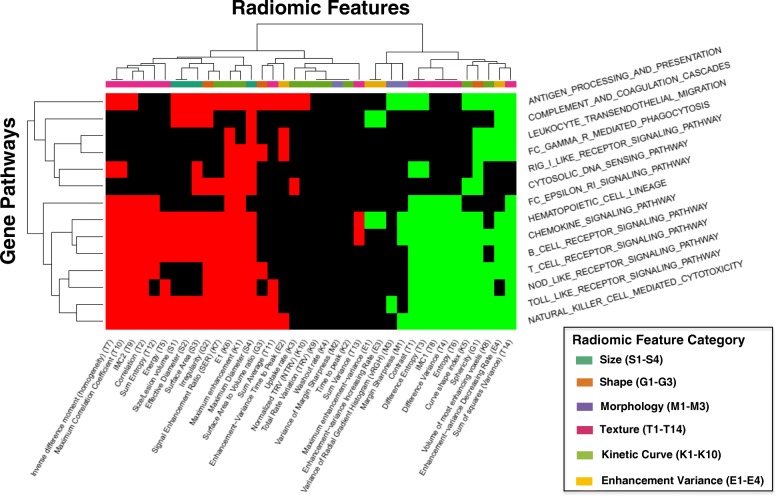
Immune Regulation**.** Pathways involved in immune regulation that have at least one significant positive (green) or negative (red) association with a radiomic features are included in this diagram. These pathways include a broad array of cascades: antigen processing and presentation, complement and coagulation, leukocyte migration, IgG mediated phagocytosis, RIG1-like receptor, cytosolic DNA sensing, IgE receptor, hematopoiesis, chemokine signaling, B and T-cell signaling, NOD-like and toll-like receptors, and NK cell mediated cytotoxicity. Radiomic features are classified by the six characteristic categories as described previously

Out of all pathway categories, those related to immune regulation had the highest number of significant correlations with radiomic features, with an average of 18.9 associations per pathway (Fig. 2). A diverse set of immune pathways was found to be associated with several imaging features, ranging from adaptive immune responses such as B and T-cell signaling to more innate immune responses such as toll-like and NOD-like receptor signaling and complement activation (Fig. 4). Notably, the directionality of association for the immune pathways were the same across individual imaging phenotypes, suggesting that these pathways, which cover many different types of immune cells and functional components of the immune system, influence the appearance of the tumor on the MRI in a similar fashion. Overall, higher immune pathway activation was associated with smaller size, increased sphericity, shaper margins, and more variable texture on contrast imaging, all indicating better prognosis.

### Cell-cell interaction

**Fig. 5:**
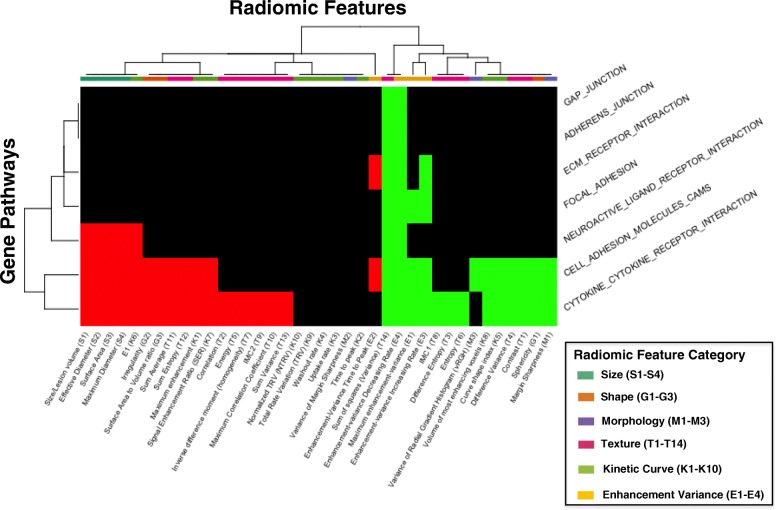
Cell-cell Interaction**.** Pathways involved in cell-cell interaction that have at least one significant positive (green) or negative (red) association with a radiomic features are included in this diagram. These pathways include the gap and adherens junctions, extracellular matrix receptors, focal adhesion, cell adhesion molecules, and cytokine-cytokine receptor cascades. Radiomic features are classified by the six characteristic categories as described previously

Pathways related to extracellular signaling molecules had the second highest number of significant correlations, with an average of 16 associations per pathway (Fig. 2). We compared these pathways to those involved in direct epithelial cell-cell signaling, which only had an average of 2.7 associations per pathway. Of the pathways representing these interactions, the two that had the most robust association with radiomic features include the cell adhesion molecule (CAM) signaling and the cytokine-cytokine receptor signaling cascades (Fig. 5). All size phenotypes were negatively correlated with these two pathways. All shape phenotypes were also significantly associated with these two cascades, with positive associations seen with sphericity and negative associations with irregularity and surface area to volume ratio. These correlations suggested that tumors that had higher CAM and cytokine signaling events tended to be smaller, more spherical, and less irregular in shape. All enhancement texture features (T1-T14) were significantly associated with the cytokine cascade. Notably, positive correlations with contrast, difference variance, entropy, and negative correlations with image linearity and energy (image homogeneity) suggest that higher cytokine signaling was associated with more variability and less homogeneity on contrast MRI.

### Signal transduction pathways

**Fig. 6:**
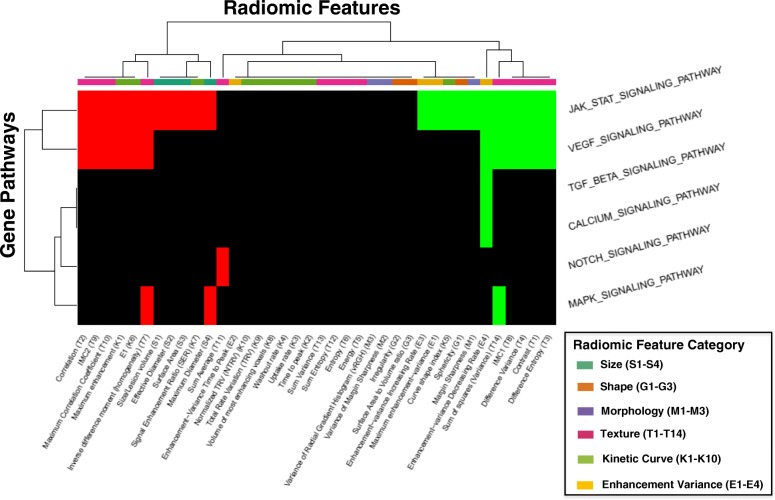
Signal Transduction**.** Pathways involved in signal transduction that have at least one significant positive (green) or negative (red) association with a radiomic features are included in this diagram. These pathways include the JAK/STAT, VEGF, TGF-Beta, calcium, notch, and MAP kinase signaling pathways. Radiomic features are classified by the six characteristic categories as described previously

We next examined the relationship between the signaling pathways and radiomic features. Six pathways had at least one significant association with a radiomic feature (Fig. 6). Two of the pathways, the VEGF and JAK/STAT signaling cascades, had the largest number of associations, with 9 of 14 enhancement texture phenotypes represented. These correlations suggested that tumors with higher expression of these two pathways appeared to have more intratumor heterogeneity in image enhancement texture: positive correlation with contrast, difference variance, and entropy, and negative correlation with homogeneity and image linearity (correlation). Of note, higher expression of the JAK/STAT pathway was also negatively correlated with all size phenotypes, whereas no such association was found with the VEGF pathway.

## Discussion

Many of our findings suggest biological plausibility and highlight interesting associations between the gene expression pathways and radiomic phenotypes that may be clinically relevant. For example, all size phenotypes were positively correlated with cell proliferation pathways and negatively correlated with the apoptosis pathway. The relationship between cell death and shape phenotypes suggests that increased expression of the apoptosis pathway was associated with more spherical and less irregular tumors. While this particular relationship is biologically predictable, it provides reassurance about the validity of using these imaging features to gain insight about others, perhaps less obvious gene expression pathways.

Perhaps most interestingly, we found that a diverse array of immune related pathways had the most robust relationship with imaging features. As a group, these pathways tended to associate with similar features in the same directionality: tumors with increased degree of overall immune activation appeared to be more confined on imaging as shown by negative correlation with size features and positive correlation with sphericity. They also tended to be more heterogeneous in texture upon contrast enhancement as seen by positive correlation with entropy and negative correlation with energy. Many of these imaging features were also similarly associated with cytokine-cytokine and CAM pathways as well as the JAK/STAT signaling cascades, which play an important role in immune regulation and in particular, lymphocyte signaling and activation. For example, Additional file 3: Figure S2C and 2D show two tumors of similar size, but the former represents a tumor with the highest level of T-cell receptor signaling activity while the latter represents a tumor with the lowest (Additional file 3: Figure S2). Whether this may in part represent an influx of tumor infiltrating lymphocytes deserves further characterization, as it may assist in the prediction of response to immune based therapies and neoadjuvant chemotherapy [45, 46] and potentially other treatment options as well.

The association between the VEGF pathway and several radiomic phenotypes was also demonstrated in our study and has a plausible mechanistic link. Dynamic contrast enhanced MRI can quantify changes in the microvascular physiology of tumors [47], and studies have shown the ability for VEGF to alter the trans-endothelial transport of contrast agent by diffusion [48]. In our study, increased expression of VEGF was positively correlated with variability of enhancement and negatively correlated with enhancement texture homogeneity, supporting the idea that VEGF activation may result in the disorganized formation of “leaky vessels” quantifiable on DCE-MRI.

This is the first report to our knowledge that integrates whole transcriptome analysis using RNASeq with 3 T DCE MRI data of patients with breast cancer. However, we acknowledge limitations of the study including the relatively small sample size leading to an inability to determine the causal relationship between the gene pathway and radiomic features. Nevertheless, the associations that we do see are biologically plausible and could lead to further advances in the analysis presented here. Several other challenges remain that must be overcome in order to better utilize the potential offered by this approach. For example, studies to date have only centered on data gathered from a single tissue sample of a given primary tumor. It is well known that the genetic makeup of tumors is spatially heterogeneous, and thus, a more accurate approach would be to correlate multiple tumor biopsies with their respective anatomic location on MRI. With techniques such as whole mount pathology being developed, it may become more feasible to pursue this idea. Also, standardization of imaging features poses a challenge. While our focus on obtaining images from one type of scanner and protocol improved the reliability of our radiomics data set, we acknowledge that image acquisition protocols also vary across institutions, and generalization of our findings will need further validation with larger sample sizes and examination of robustness studies as previously conducted for computer aided detection [39]. Lastly, as advanced MRI and other imaging techniques [49] become more widely employed, further harmonization and refinement of novel relationships may be discovered.

The recently published TCGA/TCIA Breast Phenotype Group study also combined DCE-MRI with RNA sequencing data in 91 patients, using the same quantitative radiomic approach developed by the Giger lab, although the MRIs performed in that study were acquired across several different institutions and used 1.5 T scanners. Our work further supports the feasibility of incorporating radiomics from clinical MRIs with gene expression data in support of the TCGA/TCIA Breast Phenotype Group approach using higher resolution 3 T scanners [25].

The incorporation of automated feature extraction algorithms (i.e. quantitative radiomics) into routinely performed, noninvasive imaging modalities, such as DCE-MRI has the ability to stimulate the development and use of imaging biomarkers that may provide clinicians inferred biological information otherwise attainable only through direct tissue biopsy. Potential applications include not only improvement of diagnostic ability, but also adaptation of neoadjuvant treatment plans of primary tumors in real time, such as that seen in the recent I-SPY trials [50].

## Conclusions

We provide further evidence that MR imaging of breast tumors can infer underlying gene expression using RNA sequencing data. As expected, size and shape features were appropriately correlated with proliferative and apoptotic pathways. We also discovered a large number of radiomic feature associations with immune pathways, which raises the interesting possibility of using MR imaging to distinguish tumors that are more immunologically active, although further studies are necessary to confirm this observation.

## Additional files

Additional file 1: **Table S1.** Gene Set Enrichment Analysis and Significant Pathways. The first tab includes all statistically significant associations between radiomic features and genetic pathways included in the Kyoto Encyclopedia of Genes and Genomes (KEGG) database. All these associations are identified through Gene Set Enrichment Analysis (GSEA). In the second tab, pathways are grouped by major categories and listed with the total number of radiomic associations. (XLS 129 kb)

Additional file 2: **Figure S1.** Clinicopathologic Characteristics of Patient Cases**.** Clinicopathologic parameters including age, pathologic stage, grade, histologic subtype, receptor status, as well as additional parameters involving type of surgery, chemoradiation, recurrence, and death are shown. (PPTX 61 kb)

Additional file 3: **Figure S2.** Sample 3T MR Images. Four representative cases are shown (A-D) with transverse cross-sections of breast tumors depicted in each image. Figs. C and D represents the case with the highest and lowest score with respect to T-cell receptor signaling pathway, respectively. Pathway score was obtained as follows: for each gene in a pathway, gene’s expressions were ranked over samples from high to low, and the average rank of all genes in the pathway for each sample was obtained. (PPTX 225 kb)

Additional file 4: **Figure S3.** Radiomic Feature Extraction Pipeline. Time-lapsed dynamic contrast enhanced MR images of breast cancer tissue are collected, and a staff radiologist manually indicates the tumor center. This serves as the input for computerized tumor segmentation, which delineates the boundaries for the final step, extraction of image phenotypes. Image phenotypes fall under six characteristic categories. (PPTX 79 kb)

Additional file 5: **Figure S4.** Radiomic Features by Category**.** All thirty-eight radiomic features extracted are listed according to six characteristic category including size, shape, morphology, enhancement texture, kinetic curve assessment, and enhancement-variance kinetics. (PPTX 205 kb)

## Data Availability

All clinical data analyzed and level 3 RNA sequencing data generated during this study are included in this published article (and its supplementary information files). Level 1 RNA sequencing data used during the current study is available from the corresponding author on reasonable request.
