# Supplementary material for: Radiogenomics of breast cancer using dynamic contrast enhanced MRI and gene expression profiling
Source: Cancer Imaging. 2019 Jul 15;19:48. doi: 10.1186/s40644-019-0233-5 (PMC6628478; doi:10.1186/s40644-019-0233-5)
Supplement: Supplementary file 2 — Figure S1. Clinicopathologic Characteristics of Patient Cases. Clinicopathologic parameters including age, pathologic stage, grade, histologic subtype, receptor status, as well as additional parameters involving type of surgery, chemoradiation, recurrence, and death are shown. (PPTX 61 kb) [file 40644_2019_233_MOESM2_ESM.pptx]

## Slide 1
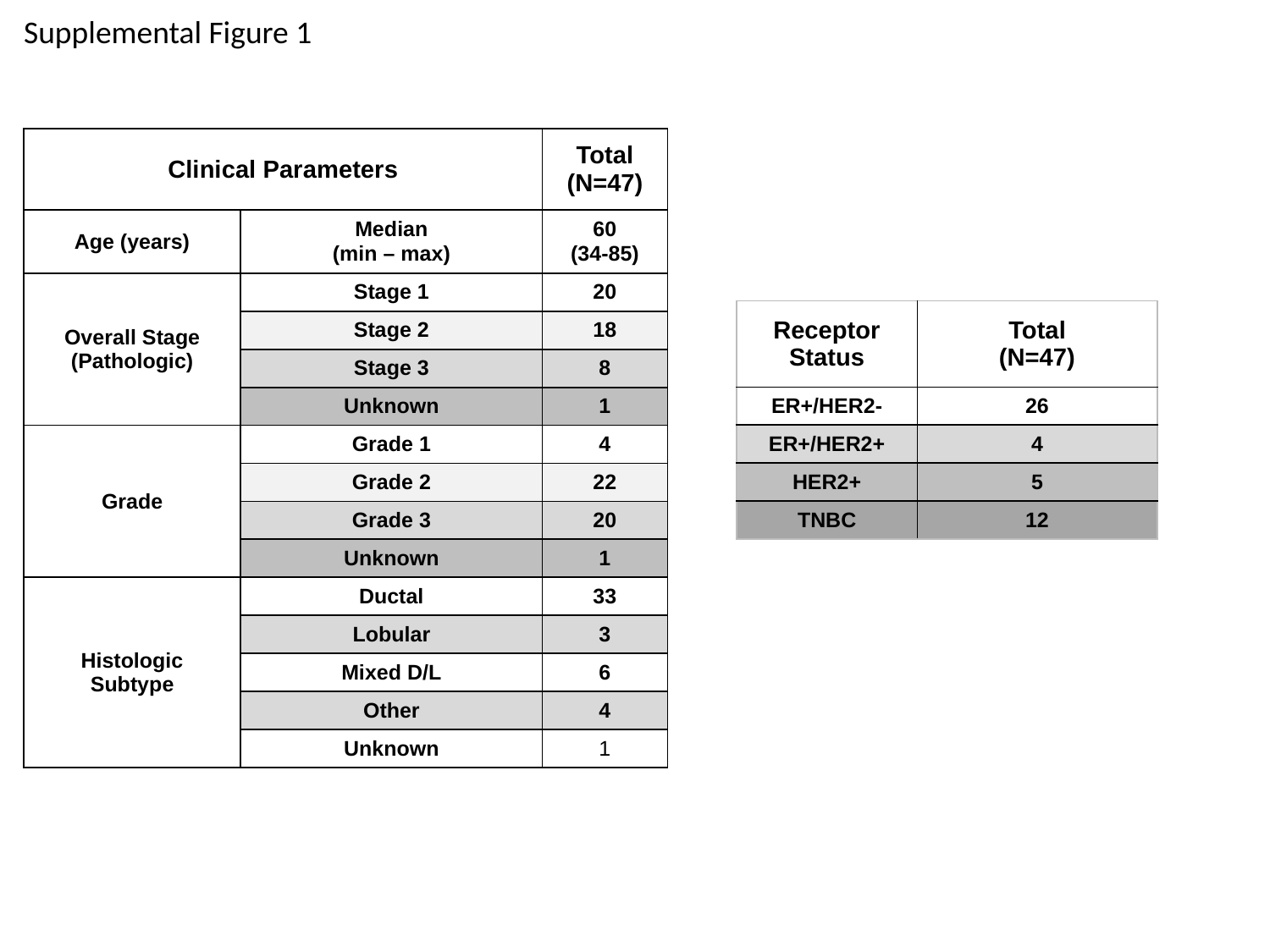

Supplemental Figure 1
| Clinical Parameters | | Total (N=47) |
| --- | --- | --- |
| Age (years) | Median (min – max) | 60 (34-85) |
| Overall Stage (Pathologic) | Stage 1 | 20 |
| | Stage 2 | 18 |
| | Stage 3 | 8 |
| | Unknown | 1 |
| Grade | Grade 1 | 4 |
| | Grade 2 | 22 |
| | Grade 3 | 20 |
| | Unknown | 1 |
| Histologic Subtype | Ductal | 33 |
| | Lobular | 3 |
| | Mixed D/L | 6 |
| | Other | 4 |
| | Unknown | 1 |
| Receptor Status | Total (N=47) |
| --- | --- |
| ER+/HER2- | 26 |
| ER+/HER2+ | 4 |
| HER2+ | 5 |
| TNBC | 12 |

## Slide 2
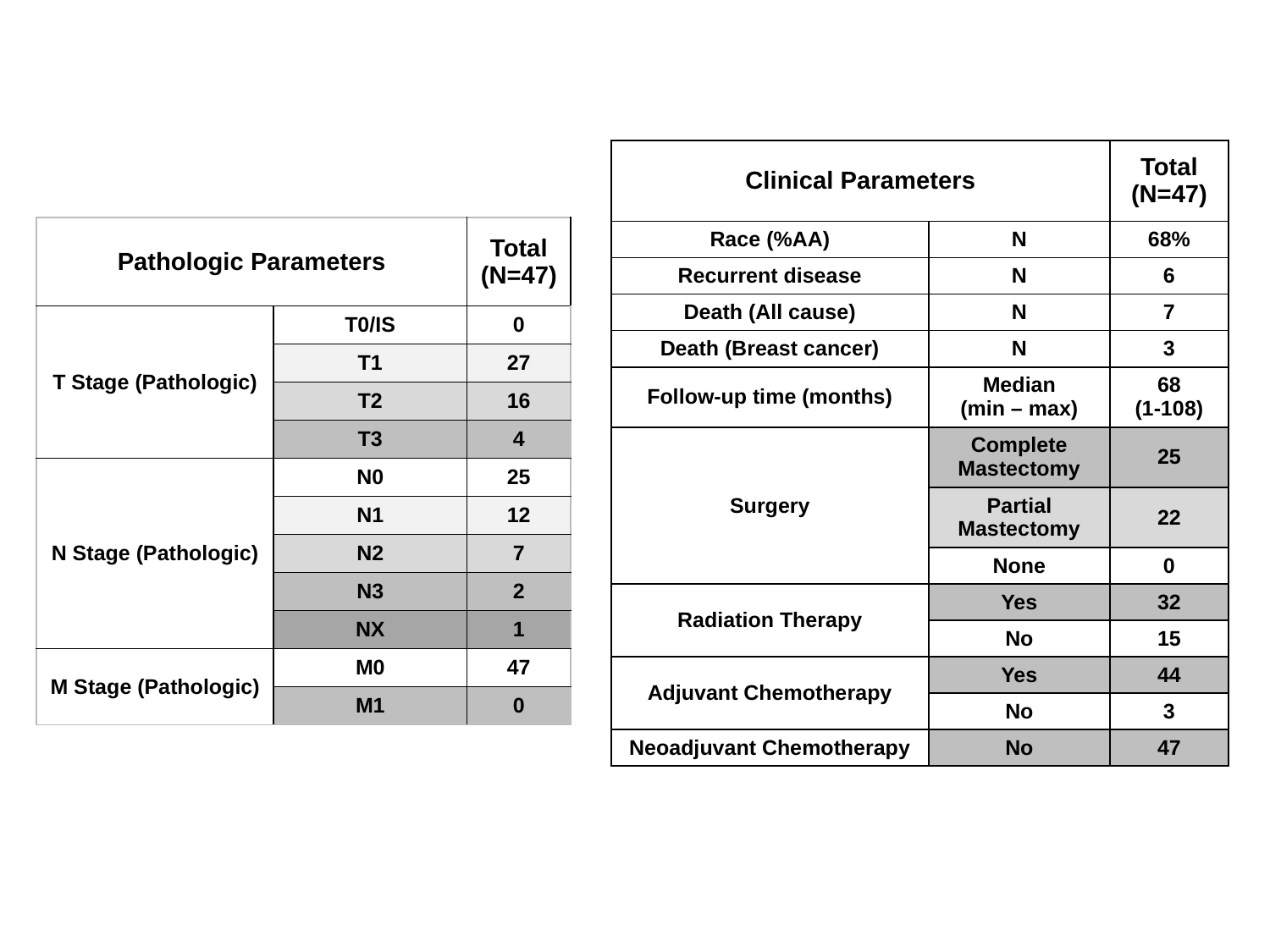

| Clinical Parameters | | Total (N=47) |
| --- | --- | --- |
| Race (%AA) | N | 68% |
| Recurrent disease | N | 6 |
| Death (All cause) | N | 7 |
| Death (Breast cancer) | N | 3 |
| Follow-up time (months) | Median (min – max) | 68 (1-108) |
| Surgery | Complete Mastectomy | 25 |
| | Partial Mastectomy | 22 |
| | None | 0 |
| Radiation Therapy | Yes | 32 |
| | No | 15 |
| Adjuvant Chemotherapy | Yes | 44 |
| | No | 3 |
| Neoadjuvant Chemotherapy | No | 47 |
| Pathologic Parameters | | Total (N=47) |
| --- | --- | --- |
| T Stage (Pathologic) | T0/IS | 0 |
| | T1 | 27 |
| | T2 | 16 |
| | T3 | 4 |
| N Stage (Pathologic) | N0 | 25 |
| | N1 | 12 |
| | N2 | 7 |
| | N3 | 2 |
| | NX | 1 |
| M Stage (Pathologic) | M0 | 47 |
| | M1 | 0 |
