# Supplementary figures and images for: Radiogenomics of breast cancer using dynamic contrast enhanced MRI and gene expression profiling
Source: Cancer Imaging. 2019 Jul 15;19:48. doi: 10.1186/s40644-019-0233-5 (PMC6628478; doi:10.1186/s40644-019-0233-5)

## Slide 1
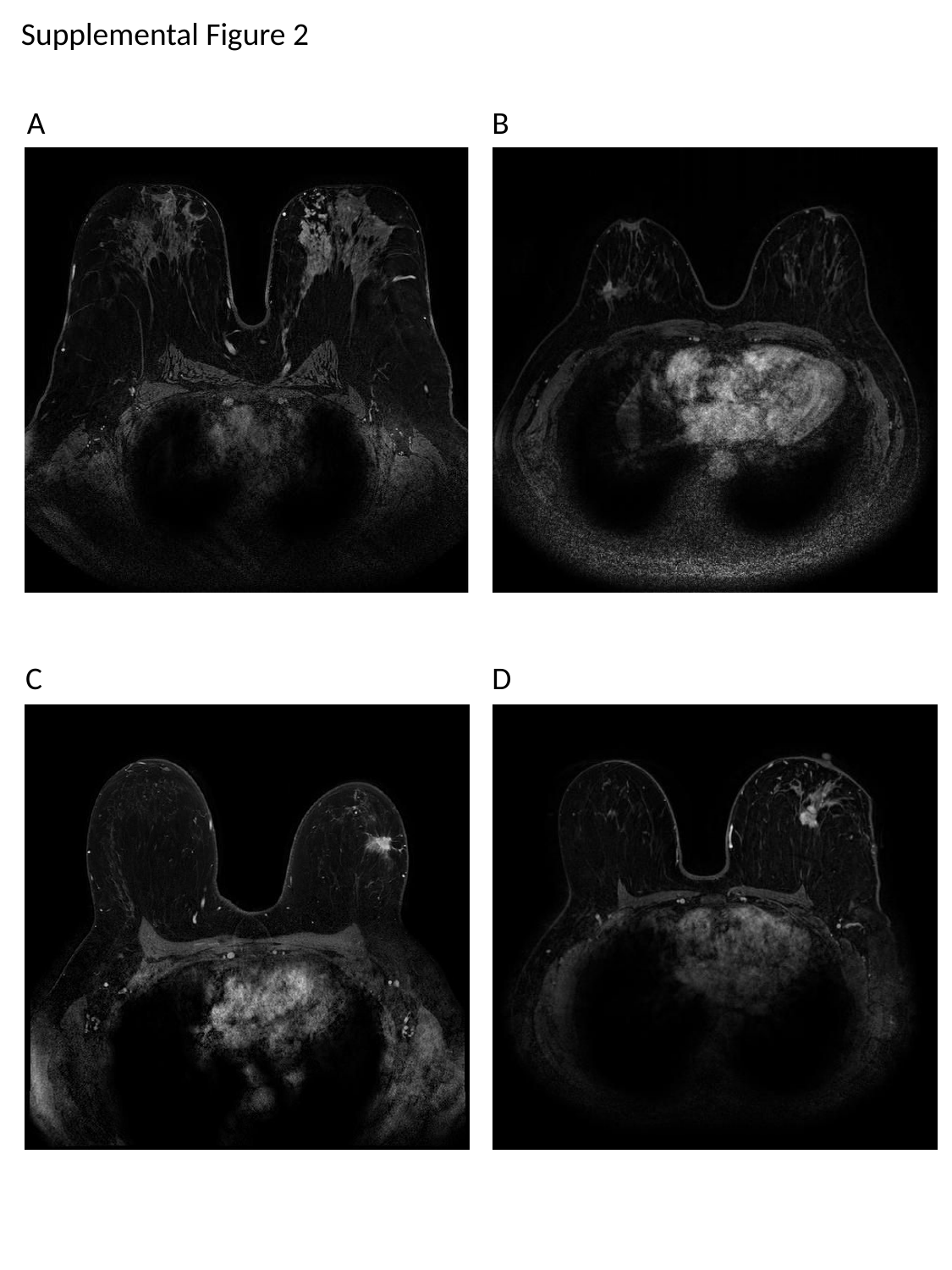

Supplemental Figure 2
A
B
C
D

Supplement: Supplementary file 3 — Figure S2. Sample 3T MR Images. Four representative cases are shown (A-D) with transverse cross-sections of breast tumors depicted in each image. Figs. C and D represents the case with the highest and lowest score with respect to T-cell receptor signaling pathway, respectively. Pathway score was obtained as follows: for each gene in a pathway, gene’s expressions were ranked over samples from high to low, and the average rank of all genes in the pathway for each sample was obtained. (PPTX 225 kb) [file 40644_2019_233_MOESM3_ESM.pptx]
