# Supplementary material for: Radiogenomics of breast cancer using dynamic contrast enhanced MRI and gene expression profiling
Source: Cancer Imaging. 2019 Jul 15;19:48. doi: 10.1186/s40644-019-0233-5 (PMC6628478; doi:10.1186/s40644-019-0233-5)
Supplement: Supplementary file 4 — Figure S3. Radiomic Feature Extraction Pipeline. Time-lapsed dynamic contrast enhanced MR images of breast cancer tissue are collected, and a staff radiologist manually indicates the tumor center. This serves as the input for computerized tumor segmentation, which delineates the boundaries for the final step, extraction of image phenotypes. Image phenotypes fall under six characteristic categories. (PPTX 79 kb) [file 40644_2019_233_MOESM4_ESM.pptx]

## Slide 1
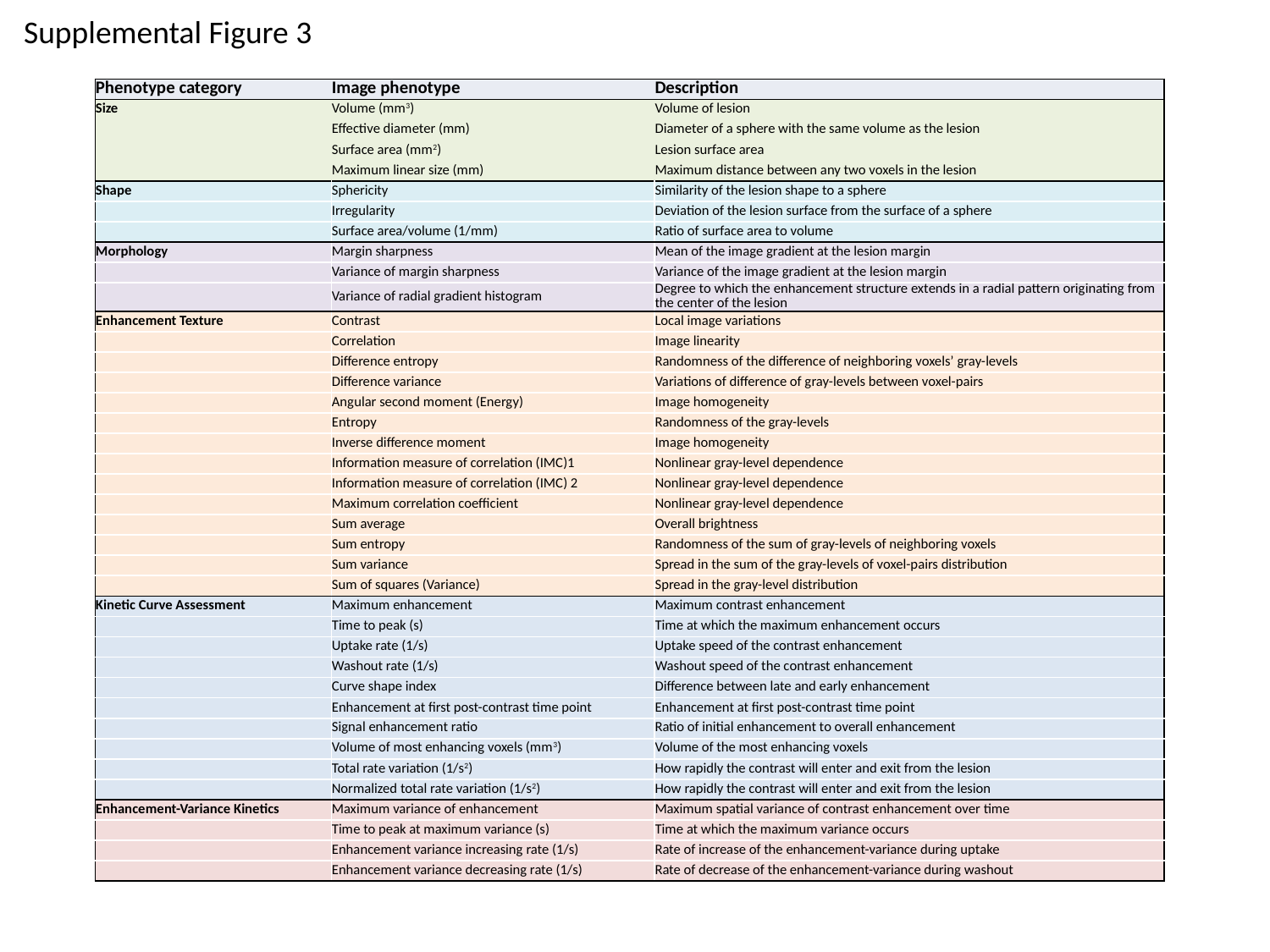

Supplemental Figure 3
| Phenotype category | Image phenotype | Description |
| --- | --- | --- |
| Size | Volume (mm3) | Volume of lesion |
| | Effective diameter (mm) | Diameter of a sphere with the same volume as the lesion |
| | Surface area (mm2) | Lesion surface area |
| | Maximum linear size (mm) | Maximum distance between any two voxels in the lesion |
| Shape | Sphericity | Similarity of the lesion shape to a sphere |
| | Irregularity | Deviation of the lesion surface from the surface of a sphere |
| | Surface area/volume (1/mm) | Ratio of surface area to volume |
| Morphology | Margin sharpness | Mean of the image gradient at the lesion margin |
| | Variance of margin sharpness | Variance of the image gradient at the lesion margin |
| | Variance of radial gradient histogram | Degree to which the enhancement structure extends in a radial pattern originating from the center of the lesion |
| Enhancement Texture | Contrast | Local image variations |
| | Correlation | Image linearity |
| | Difference entropy | Randomness of the difference of neighboring voxels’ gray-levels |
| | Difference variance | Variations of difference of gray-levels between voxel-pairs |
| | Angular second moment (Energy) | Image homogeneity |
| | Entropy | Randomness of the gray-levels |
| | Inverse difference moment | Image homogeneity |
| | Information measure of correlation (IMC)1 | Nonlinear gray-level dependence |
| | Information measure of correlation (IMC) 2 | Nonlinear gray-level dependence |
| | Maximum correlation coefficient | Nonlinear gray-level dependence |
| | Sum average | Overall brightness |
| | Sum entropy | Randomness of the sum of gray-levels of neighboring voxels |
| | Sum variance | Spread in the sum of the gray-levels of voxel-pairs distribution |
| | Sum of squares (Variance) | Spread in the gray-level distribution |
| Kinetic Curve Assessment | Maximum enhancement | Maximum contrast enhancement |
| | Time to peak (s) | Time at which the maximum enhancement occurs |
| | Uptake rate (1/s) | Uptake speed of the contrast enhancement |
| | Washout rate (1/s) | Washout speed of the contrast enhancement |
| | Curve shape index | Difference between late and early enhancement |
| | Enhancement at first post-contrast time point | Enhancement at first post-contrast time point |
| | Signal enhancement ratio | Ratio of initial enhancement to overall enhancement |
| | Volume of most enhancing voxels (mm3) | Volume of the most enhancing voxels |
| | Total rate variation (1/s2) | How rapidly the contrast will enter and exit from the lesion |
| | Normalized total rate variation (1/s2) | How rapidly the contrast will enter and exit from the lesion |
| Enhancement-Variance Kinetics | Maximum variance of enhancement | Maximum spatial variance of contrast enhancement over time |
| | Time to peak at maximum variance (s) | Time at which the maximum variance occurs |
| | Enhancement variance increasing rate (1/s) | Rate of increase of the enhancement-variance during uptake |
| | Enhancement variance decreasing rate (1/s) | Rate of decrease of the enhancement-variance during washout |
