# Supplementary material for: Radiogenomics of breast cancer using dynamic contrast enhanced MRI and gene expression profiling
Source: Cancer Imaging. 2019 Jul 15;19:48. doi: 10.1186/s40644-019-0233-5 (PMC6628478; doi:10.1186/s40644-019-0233-5)
Supplement: Supplementary file 5 — Figure S4. Radiomic Features by Category. All thirty-eight radiomic features extracted are listed according to six characteristic category including size, shape, morphology, enhancement texture, kinetic curve assessment, and enhancement-variance kinetics. (PPTX 205 kb) [file 40644_2019_233_MOESM5_ESM.pptx]

## Slide 1
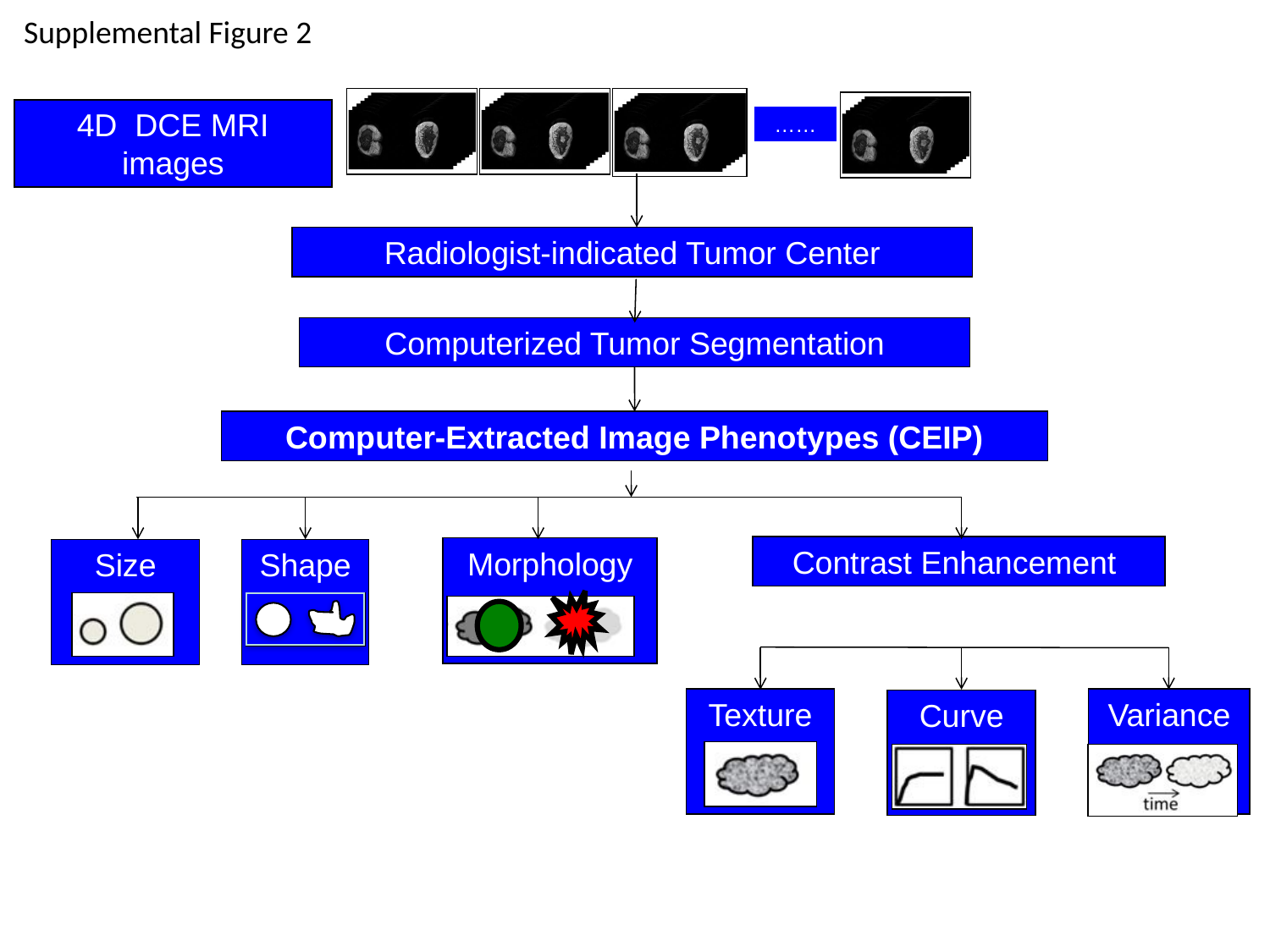

Supplemental Figure 2
4D DCE MRI images
……
Radiologist-indicated Tumor Center
Computerized Tumor Segmentation
Computer-Extracted Image Phenotypes (CEIP)
Contrast Enhancement
Morphology
Size
Shape
Texture
Variance
Curve
